# The presence of two rare genomic syndromes, 1q21 deletion and Xq28 duplication, segregating independently in a family with intellectual disability

**DOI:** 10.1186/s13039-016-0286-0

**Published:** 2016-09-29

**Authors:** Kyungsoo Ha, Yiping Shen, Tyler Graves, Cheol-Hee Kim, Hyung-Goo Kim

**Affiliations:** 1 Department of Molecular Physiology and Biophysics, Baylor College of Medicine, Houston, TX 77030 USA; 2 Section of Reproductive Endocrinology, Infertility & Genetics, Department of Obstetrics & Gynecology, Augusta University, Augusta, GA 30912 USA; 3 Department of Neurology, Massachusetts General Hospital, Harvard Medical School, Boston, MA 02114 USA; 4 Department of Biology, Chungnam National University, Daejeon, 34134 South Korea; 5 Department of Neuroscience and Regenerative Medicine, Augusta University, 1120 15th Street, Augusta, GA 30912 USA

**Keywords:** Intellectual disability, 1q21 microdeletion, MECP2, Xq28 duplication, X chromosome inactivation, Segregation of two rare syndromes

## Abstract

**Background:**

1q21 microdeletion syndrome is a rare contiguous gene deletion disorder with *de novo* or autosomal dominant inheritance patterns and its phenotypic features include intellectual disability, distinctive facial dysmorphism, microcephaly, cardiac abnormalities, and cataracts. *MECP2* duplication syndrome is an X-linked recessive neurodevelopmental disorder characterized by intellectual disability, global developmental delay, and other neurological complications including late-onset seizures. Previously, these two different genetic syndromes have not been reported segregating independently in a same family.

**Case presentation:**

Here we describe two siblings carrying either a chromosome 1q21 microdeletion or a chromosome Xq28 duplication. Using a comparative genomic hybridization (CGH) array, we identified a 1.24 Mb heterozygous deletion at 1q21 resulting in the loss of 9 genes in a girl with learning disability, hypothyroidism, short stature, sensory integration disorder, and soft dysmorphic features including cupped ears and a unilateral ear pit. We also characterized a 508 kb Xq28 duplication encompassing *MECP2* in her younger brother with hypotonia, poor speech, cognitive and motor impairment. The parental CGH and quantitative PCR (qPCR) analyses revealed that the 1q21 deletion in the elder sister is *de novo*, but the Xq28 duplication in the younger brother was originally inherited from the maternal grandmother through the mother, both of whom are asymptomatic carriers. RT-qPCR assays revealed that the affected brother has almost double the amount of *MECP2* mRNA expression compared to other family members of both genders including maternal grandmother and mother who have the same Xq28 duplication with no phenotype. This suggests the X chromosome with an Xq28 duplication in the carrier females is preferentially silenced.

**Conclusion:**

From our understanding, this would be the first report showing the independent segregation of two genetically unrelated syndromes, 1q21 microdeletion and Xq28 duplication, in a same family, especially in siblings. Although these two chromosomal abnormalities share some similar phenotypes such as intellectual disability, mild dysmorphic features, and cardiac abnormalities, the presence of two unrelated and rare syndromes in siblings is very unusual. Therefore, further comprehensive investigations in similar cases are required for future studies.

## Background

The 1q21 microdeletion (MIM 612474) is a very rare genetic disorder with the deleted region spanning from 145.4 Mb to 147.8 Mb (hg19) on chromosome 1. 1q21 microdeletion can occur *de novo*, but the majority of microdeletions are inherited from either parent in an autosomal dominant manner [1–3]. Haploinsufficiency of the genes deleted at 1q21 likely contribute to the clinical phenotypes associated with this microdeletion. A parent with the same microdeletion generally shows a normal phenotype, or very mild phenotype that is similar to or less severe than that of their child [1]. This suggests the possibility of parental imprinting, reduced penetrance, or variable expressivity. The phenotypic features prompting diagnosis of 1q21 microdeletion include mild to moderate intellectual disability, learning disability, microcephaly, mildly dysmorphic facial features and skeletal malformations [1–4], cataracts [2, 5] and heart abnormalities [5, 6]. Because these clinical features are also seen in other genetic disorders, 1q21 microdeletion is difficult to be diagnosed clinically and more often molecularly diagnosed by chromosomal microarray analysis [1, 2, 4].

Xq28 duplication syndrome (MIM 300815) resulting in functional disomy of Xq28 has been predominantly described in male patients with syndromic intellectual disability including infantile hypotonia, poor or absent speech, and recurrent pulmonary infections [7, 8]. The clinical features of Xq28 duplications differ, dependent on the location and the size ranging typically from 0.3 to 2 Mb [9–12]. One of the multiple genes duplicated in this intellectual disability syndrome is methyl CpG-binding protein 2 (*MECP2*; MIM 300005), a chromosomal protein that binds specifically to methylated DNA [13]. Early studies demonstrated that MECP2 represses transcription from methylated promoters [14] and is subject to X chromosome inactivation in both mouse and human [15, 16]. Furthermore, MECP2 is an essential regulator in postnatal brain development and a critical dosage-sensitive gene involved in neurological abnormalities [17, 18]. In the inherited cases, the disorder almost exclusively affects males, and the MECP2 duplication in a carrier mother with favorable skewing of X-chromosome inactivation results in minimal to no clinical findings [8] But rarely, females who carry the same duplication on one X chromosome may exhibit mild to severe clinical features seen in affected males [12, 19]. In some cases, the duplication occurs *de novo* [12, 20].

Here we report two genetically unrelated rare syndromes in a family. A deletion in a girl and one duplication inherited in her brother. The first chromosomal aberration is a *de novo* 1q21 microdeletion involving 9 genes. The other chromosomal anomaly, Xq28 duplication encompassing 15 genes including *MECP2*, is inherited from the asymptomatic mother. We performed qPCR to further confirm the CNVs and familial inheritance patterns of these chromosome rearrangements, and quantified the mRNA expression levels of *MECP2* using RT-qPCR. In addition, we discussed genotype-phenotype correlations in patients with these two rare syndromes.

## Methods

### Cell culture

Lymphoblastoid cell lines (LCLs) were established from blood samples obtained from patients and their family members and maintained as previously described [21].

### Comparative genomic hybridization (CGH) array

Genomic DNA (gDNA) was extracted from peripheral blood lymphocytes using standard phenol/chloroform extraction method [21]. The isolated genomic DNAs were examined for copy number variations (CNVs) using the Agilent 244 K Agilent comparative genomic hybridization (CGH) array (Agilent Technologies).

### Quantitative polymerase chain reaction (qPCR) for the confirmation of deletion and duplication

Primers used for the confirmation of the 1q21 and 7q11 deletion and Xq28 duplication are listed in Table 1. A primer set amplifying the exon 8 of *GAPDH* was used as an internal control. Total 10 ng of gDNA was mixed with Power SYBR Green PCR Master Mix (Applied Biosystems, Carlsbad, CA) and the primers, according to the manufacturer’s protocol. Quantitative PCR (qPCR) was performed for the amplification of the targeted region utilizing a 7500 Real-Time PCR system (Applied Biosystems, Carlsbad, CA). The PCR reactions were cycled 40 times after initial denaturation (95 °C, 10 min) with the following parameters: denaturation at 95 °C for 15 s; annealing at 60 °C for 1 min. The amplification levels of *GAPDH* exon 8 were used to normalize relative levels of DNA.

**Table 1:** Primers used for qPCR in this study

Name	Primer sequence (5’ → 3’)	
	Forward	Reverse
chr1q21.1 #1	TGAGCAGTTCAAAGGAGTGTAG	ATGACCCACAAAGTGAGAGAAA
chr1q21.1 #2	AAGGCTGTGAAGGAGGAAATC	CTGACCAGGCAGAAGACATAAA
chr7q11	GGAGCACAAAGCAACTGAATG	AGACAGCAATGCAGAGGAA
chrXq28 #1	AGAGCTCGGACTCCATCTAAT	CCTTCCCATGTCAGTGTGTTAT
chrXq28 #2	CTCACTTCTGGGTCTCACATTC	AATCCCAAGTGACTTCCAAGG
GAPDH	GATCATCAGCAATGCCTCCT	ATGGCATGGACTGTGGTCAT

### Reverse transcription-quantitative polymerase chain reaction (RT-qPCR)

Total RNA was extracted from the lymphocyte cell lines using RNeasy Mini Kit (Qiagen, Valencia, CA) according to the instructions of the manufacturer. Total RNA 2 μg was reverse transcribed and converted into complementary DNA (cDNA) using the RevertAid First Strand cDNA synthesis kit (Thermo Scientific, Waltham, MA). The following are primer sequences used for amplification of *MECP2* (NM_004992.3) and *GAPDH* (NM_002046.4); *MECP2 #1* forward 5’ – CTCAGGCCATTCCCAAGAAA – 3’ and reverse 5’ – TCCTGCACAGATCGGATAGA – 3’; *MECP2 #2* forward 5’ – CTCTGCTGGGAAGTATGATGTG – 3’ reverse 5’ – TCATTAGGGTCCAGGGATGT – 3’; *GAPDH* forward 5’ – GATCATCAGCAATGCCTCCT – 3’ and reverse 5’ – ATGGCATGGACTGTGGTCAT – 3’. The cDNAs were mixed with Power SYBR Green PCR Master Mix (Applied Biosystems, Carlsbad, CA) and primers. All samples and loading controls were plated in triplicate and centrifuged briefly. RT-qPCR was performed for the amplification of *MECP2* and *GAPDH* utilizing a 7500 Real-Time PCR system (Applied Biosystems, Carlsbad, CA). The PCR reactions were cycled 40 times after initial denaturation (95 °C, 10 min) with the following parameters: denaturation at 95 °C for 15 s; annealing at 60 °C for 1 min. Expression of *GAPDH* was used to normalize relative expression of *MECP2* mRNA.

## Case presentation

**Fig. 1:**
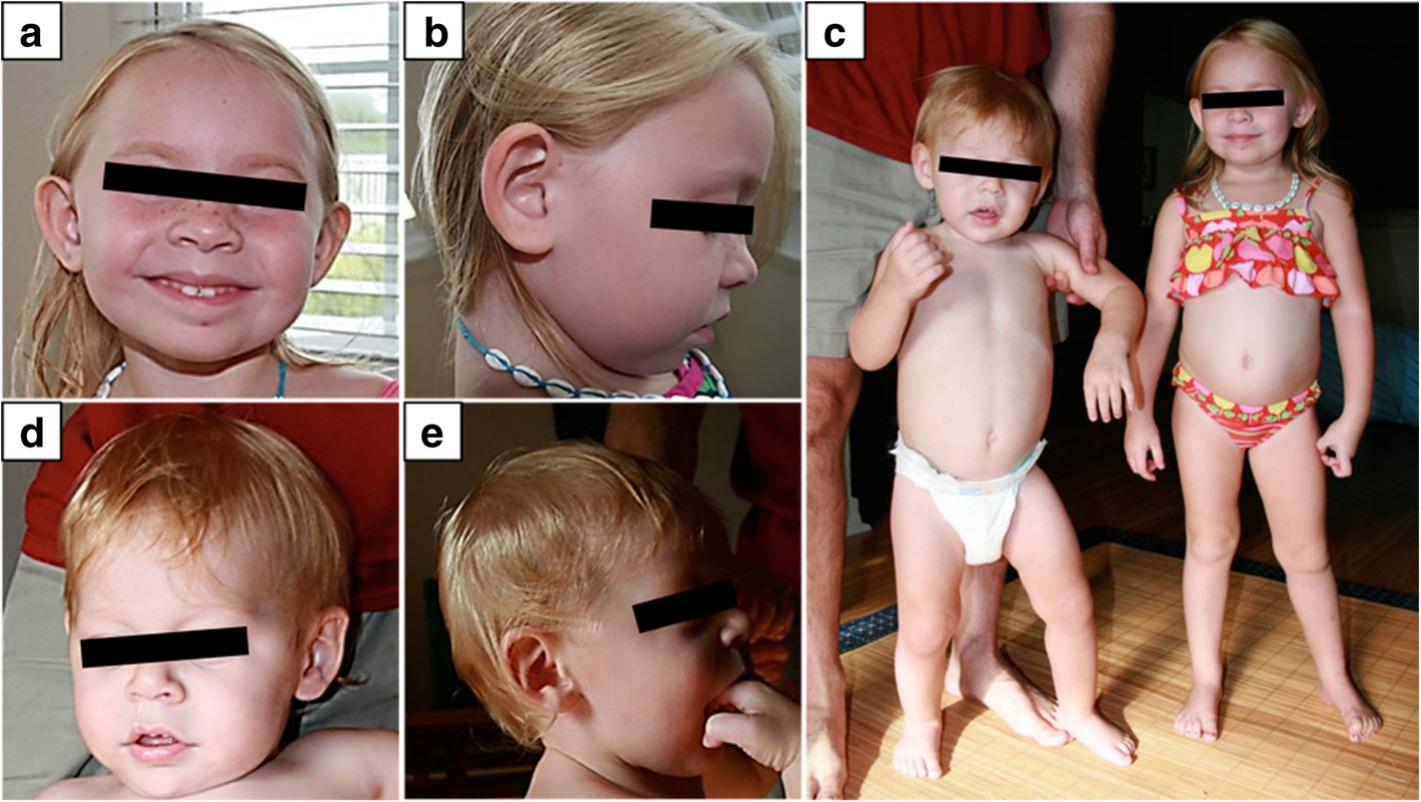
Facial and whole body photographs of two affected children in our study. **a**, **b** Proband 1 at age of 4 years and 8 months has cupped ears as well as a unilateral ear pit in her right ear. **c** Proband 1 at age of 4 years and 8 months shows similar length with her younger brother, proband 2 at 2 years and 2 months. **d**, **e** Proband 2 at age of 2 years and 2 months has mild craniofacial dysmorphisms with bilateral epicanthal folds and periorbital swelling

Our proband 1 is a 10 and half-year old Caucasian female with learning disability, significant growth retardation, microcephaly and speech delay. She was born at a full term after a normal pregnancy and delivery with average birth weight (3.27 kg, 50 percentile) and length (49.5 cm, 50 percentile). No jaundice, hypotonia or problems with feeds was observed during newborn period. However, her weight was 25th percentile at 2 months, and less than 3rd percentile from age 8 months. Although both her weight and length has been below 3rd percentile from age of 9 months, she has not shown any sign of delayed milestones. At the age of seven months, she developed oral thrush and began to demonstrate decreased development and feeding. She became a poor eater and tended to only drink water and breastfeed for liquids. She had failure to thrive partly because of her swallowing problem and tongue thrust. Since 6 months old, she continues to have constipation with bowel movement every 3–5 days. It has not improved with thyroid treatment and enema was required at times. At 1 year and 10 months, she had laboratory evidence of compensated hypothyroidism with normal thyroid levels and elevated thyroid stimulating hormone, on Synthroid treatment for symptomatic control. She has soft dysmorphic features including cupped ears and a unilateral ear pit in her right ear (Fig. 1a, b). Her length was below the 3rd percentile, but her weight to length curve was normal in the 22nd percentile (Fig. 1c). Brain MRI, performed at 2 years and 3 months of age, showed normal. She presents significant overall sensory regulation issues. For instance, she seeks movement and touch constantly and has inconsistent sleep patterns as well. She demonstrates behaviors consistent with anxiety and high arousal state. She seeks specific oral motor input and prefers to have crunch and/or chewy textures. Her behavioral studies revealed the auditory processing concerns with difficulty processing specific directions, which are consistent with a system that is high in adrenaline and low in serotonin and dopamine. She has a learning disability, struggling in math at the school, and is seeing a speech therapist for reading difficulties. At 10.5 years of age, her cognitive function is at the level of 8.5 years.

**Fig. 2:**
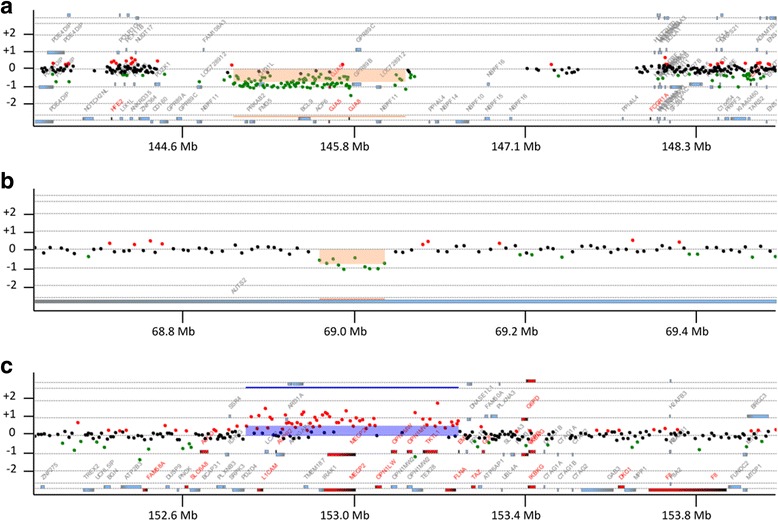
The relevant section of copy number variants (CNVs) by array comparative genomic hybridization with the use of Agilent 244 K arrays. Please note that the coordinates shown in Fig. 2 are based on NCBI36/hg18 of the Human Genome Browser, which were translated into GRCh37/hg19 in the Case Presentation section. **a** 1q21 deletion in proband 1. **b** 7q11.22 deletion in proband 1. **c** Xq28 duplication in proband 2. On the scale of deviation from the normal diploid genotype, −2 indicates a homozygous deletion, −1 indicates a haploid deletion, 0 indicates no deviation, 1 indicates a duplication, and 2 indicates a triplication. X axis indicates the location of CNVs on chromosomes (hg18)

**Fig. 3:**
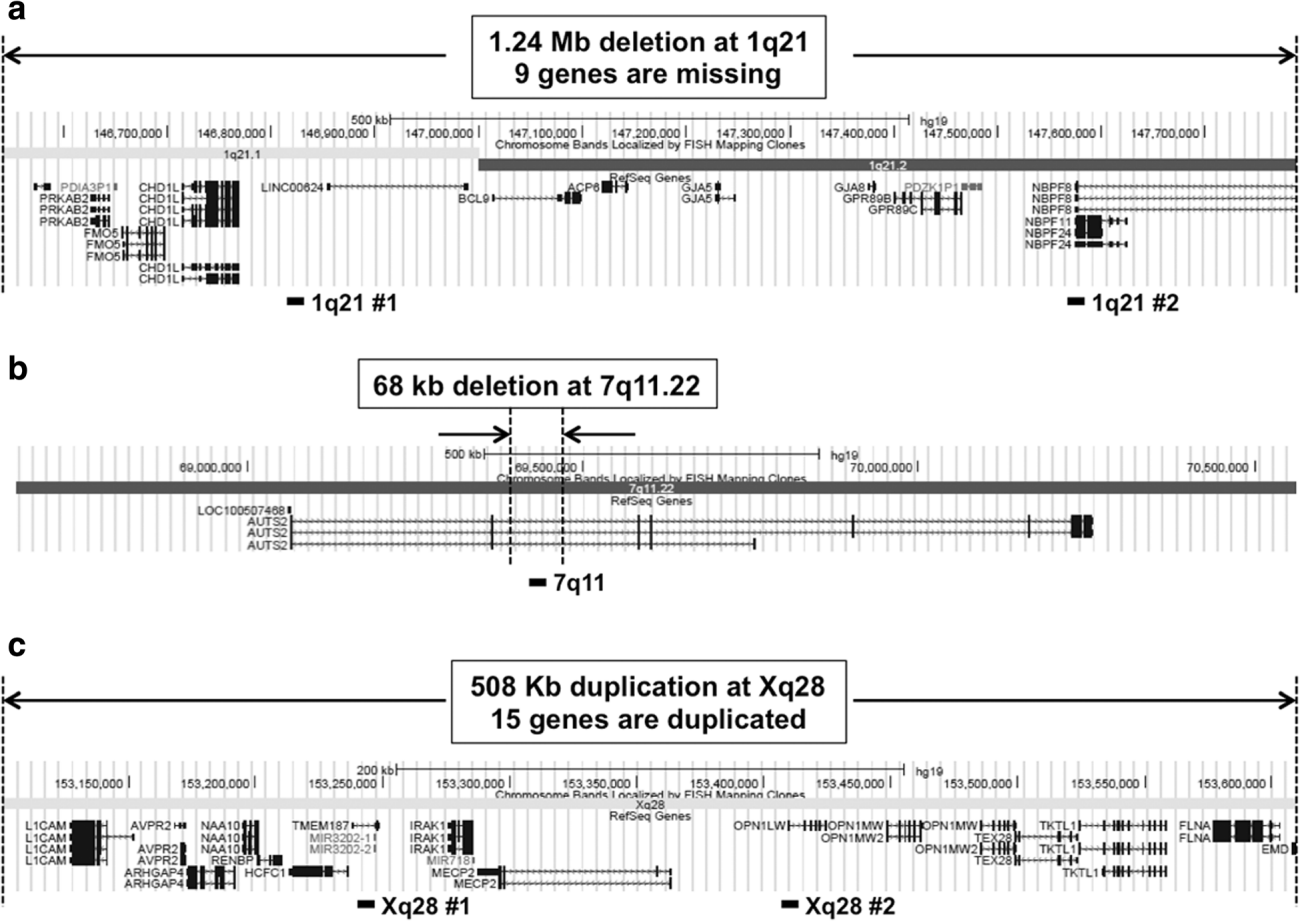
Genome view of deletion and duplication regions in proband 1 (**a** and **b**) and 2 (**c**) exported from Human Genome Browser (Build 37/hg19). RefSeq genes are described in http://www.genome.ucsc.edu/. Black boxes under browser maps show approximate locations of loci where primers were designed for qPCR

**Fig. 4:**
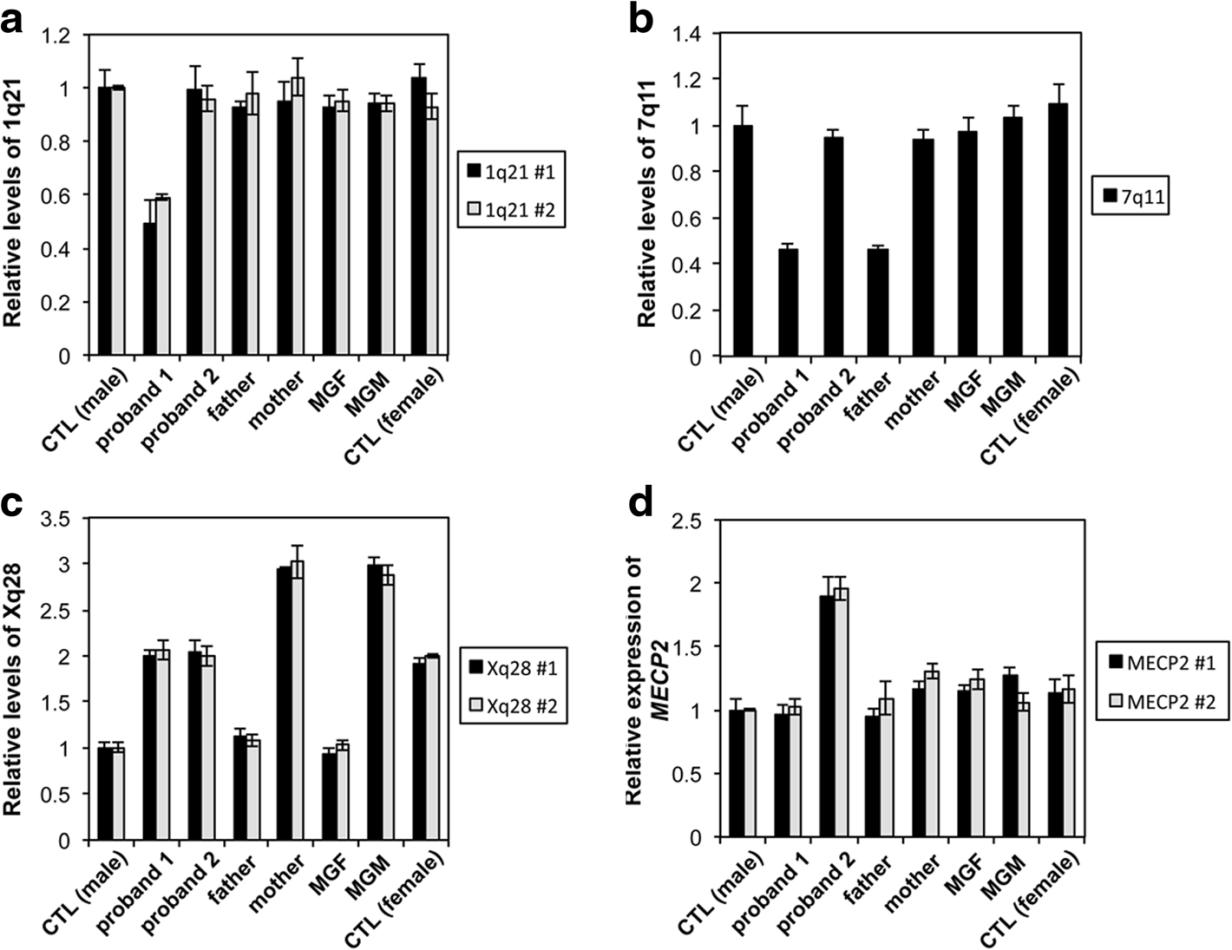
Quantitative PCR results of deletions and duplication in our family members. **a** qPCR results showing the 1q21 deletion using 2 different set of primers amplifying the regions within the known deleted region in proband 1. Deletion was detected only in proband 1. A value close to 0.5 indicates deletion on one chromosome 1, but a value close 1 indicates no deletion. **b** qPCR results showing the 7q11 deletion in both father and proband 1. Deletion in proband 1 was inherited from healthy father suggesting that this deletion is polymorphism. A value close to 0.5 indicates deletion on one chromosome 7, but a value close 1 indicates no deletion. **c** qPCR results showing duplication of Xq28 in proband 2, mother and MGM. Xq28 duplication in proband 2 is originally inherited from the maternal grandmother. Values indicate that: 1 (no deletion on chromosome X in male); 2 (duplication on chromosome X in male, no duplication in female); 3 (duplication on chromosome X in female). The amplification levels of *GAPDH* exon 8 were used to normalize relative levels of DNA. **d** The mRNA expression levels of *MECP2* in the family members. Quantitative RT-PCR was performed to measure the mRNA levels of *MECP2* using 2 different sets of primers specific to *MECP2* mRNA (NM_004992.3). Expression of *GAPDH* was used to normalize relative expression of *MECP2* mRNA. Error bars represent standard errors. Proband 1: a 10-year old Caucasian female with 1q21 microdeletion; Proband 2: an 8-year old Caucasian male with Xq28 duplication; MGF: maternal grandfather; MGM: maternal grandmother

A 244K Agilent CGH array revealed a deletion of 1q21.1-1q21.2 spanning about 1.24 Mb of genomic DNA with the genome coordinates from 146,542,843 to147,786,706 (GRCh37/hg19), resulting in the loss of 9 genes including *PRKAB2, FMO5, CDH1L, BCL9, ACP6, GJA5, GJA8, GPR89B, and NBPF11* (Figs. 2a and 3a, Table 2). This deletion has been reported to be causative for the 1q21 recurrent microdeletion syndrome [2]. The results of CGH array in both parents demonstrated that this del(1)(q21.1q21.2) in proband 1 occurred *de novo* and this result was also confirmed by qPCR (Fig. 4a). Another deletion identified in proband 1 was a 68 Kb deletion within intron 2 of *AUTS2* gene at 7q11.22 (Figs. 2b and 3b). The qPCR results showed that the intronic deletion in AUTS2 in proband 1 was inherited from the healthy father suggesting that this deletion is a polymorphism with no clinical effect (Fig. 4b).

**Table 2:** List of affected genes in probands 1 and 2

Gene symbol	Gene name	OMIM #	Function
Proband 1 with 1.24 Mb deletion at 1q21			
*PRKAB2*	AMP-activated kinase complex noncatalytic beta-2	602741	Maintains systemic and cellular energy homeostasis
*FMO5*	Flavin-containing monooxygenase 5	603957	Involved in the metabolic activation of drugs and xenobiotic compounds
*CHD1L*	Chromodomain helicase DNA-binding protein 1-like	613039	Has a role in chromatin modification and DNA damage response
*BCL9*	B-cell CLL/lymphoma 9	602597	A signal transduction protein required for efficient beta-catenin-mediated transcription in Wnt signaling pathway
*ACP6*	Acid phosphatase 6	611471	Hydrolyzes lysophosphatidic acid containing fatty acid
*GJA5*	Gap junction protein, alpha-5	121013	A cardiac gap junction protein connexin 40 that facilitates cell-to-cell adhesion and intercellular communication
*GJA8*	Gap junction protein, alpha-8	600897	A transmembrane connexin protein that is necessary for lens growth and maturation of lens fiber cells
*GPR89B*	G protein-coupled receptor 89B	612806	Voltage dependent anion channel regulating the acidification and function of Golgi apparatus
*NBPF11*	Neuroblastoma breakpoint family, member 11	614001	A member of the NBPF family and diseases associated with *NBPF11* include neuroblastoma
Proband 2 with 508 kb duplication at Xq28			
L1CAM	L1 cell adhesion molecule	308840	Belongs to immunoglobulin superfamily cell adhesion molecules and has a role in neuronal migration and survival
AVPR2	arginine vasopressin receptor 2	300538	G protein-coupled receptor involved in the regulation of the urine and water homeostasis in kidney
ARHGAP4	Rho GTPase activating protein 4	300023	Regulates the function of small GTP-binding proteins belonging to the RAS superfamily
NAA10	N(alpha)-acetyltransferase 10, NatA catalytic subunit	300013	Catalytic subunit of the N-terminal acetyltransferase A complex, which transfers an acetyl group from acetyl-coenzyme A to the alpha-amino group on a nascent polypeptide
RENBP	renin binding protein	312420	Inhibits renin by forming a dimer with renin and involved in transport to the Golgi and synthesis of substrates in N-glycan biosynthesis
HCFC1	host cell factor C1	300019	Involved in cell cycle regulation and functions as a transcription repressor by inhibiting the recruitment of p300 to promoter. Mutations of this gene cause non-syndromic X-linked intellectual disability and X-linked cobalamin disorder.
TMEM187	transmembrane protein 187	300059	A multi-pass membrane protein, but its biological function is not determined
IRAK1	interleukin 1 receptor associated kinase 1	300283	A putative serine/threonine kinase that plays a critical role in immune response and become associated with the IL-1 receptor
MECP2	methyl-CpG binding protein 2	300005	Specifically binds to a single methyl-CpG pair and mediates transcriptional repression through interaction with histone deacetylase and a corepressor
OPN1LW	opsin 1 (cone pigments), long-wave-sensitive	300822	Long-wavelength sensitive opsin, transmembrane receptor protein with a visual pigment, which is a light-absorbing molecules that mediate vision
OPN1MW	opsin 1 (cone pigments), medium-wave-sensitive	300821	Medium-wavelength sensitive opsin, transmembrane receptor protein with a visual pigment, which is a light-absorbing molecules that mediate vision
TEX28	testis expressed 28	300092	A member of the red/green cone visual pigment gene family
TKTL1/TKT2	transketolase-like 1/transketolase 2	300044	A thiamine-dependent enzyme that links the pentose phosphate pathway with the glycolytic pathway.
FLNA	Filamin A	300017	An actin-binding protein involved in the reorganization of cytoskeletion to effect in cell migration
EMD	emerin	300384	A nuclear membrane protein that associates with the nuclear membrane lamina and mediates membrane anchorage to the cytoskeleton

Proband 2 is a 8-year old Caucasian male with developmental delays, hypotonia, speech delay as well as cognitive and motor impairment. He was born at full term following a normal pregnancy, labor and delivery with appropriate growth parameters, birth weight 3.92 Kg and length 51 cm. He remained in a hospital for three days due to a detected ventricular septal defect (VSD) and breathing problems. Later, he was admitted two times at the ages of four months and then fourteen months for enlarged heart and cyanotic changes, but VSD subsequently closed spontaneously. At the age of eleven months, he had pressure equalization tubes and was diagnosed with juvenile idiopathic arthritis. His developmental delay particularly regarding his motor milestones was observed from 6 months of age; rolling from front to back and back to front at 3 months of age; pulling to stand at 10 months; sitting at 11 months; crawling in reciprocal fashion by 13 months of age and walking with support at 20 months. However, muscle mass, strength and muscle stretch reflexes were within normal limits. There is no history of seizures or periodic breathing, and no major difficulties with constipation or teeth grinding. In the developmental test performed at the age of 2 years, he demonstrated severe delays across all areas consistent with overall cognitive and motor impairment as well as craniofacial dysmorphisms with bilateral epicanthal folds and periorbital swelling (Fig. 1d, e). He had a few mild behaviors consistent with an autistic spectrum disorder such as lack of pointing, limited play skills and poor eye contact, but generally seems to interact at a level consistent with his developmental level. His poor eye contact also seems more a symptom of overall poor attention span, and visual inattentiveness, as opposed to lack of social awareness. He also displayed limited integration of social communication behaviors (gaze, sounds, gestures and facial expressions) and difficulty initiating and sustaining social interactions.

A CGH array result demonstrated that proband 2 has 508 Kb duplication at Xq28 with the genome coordinates from 153,101,077 to 153,609,163 (GRCh37/hg19) encompassing 15 genes including *MECP2*, one of responsible genes for Xq28 duplication syndrome (Figs. 2c and 3c, Table 2). Parental CGH array results revealed that this Xq28 duplication was inherited from mother. The qPCR results using two different primers amplifying regions within the known duplicated region revealed that Xq28 duplication was originally inherited from maternal grandmother who inherited it to mother, a carrier with a normal phenotype (Fig. 4c). We performed qRT-PCR to compare the mRNA expression levels of *MECP2* in proband 2 with those in other family members whose blood samples were available. Figure 4d shows proband 2 has almost double amount of *MECP2* mRNA expression compared to other family members. However, both maternal grandmother and mother have normal expression levels of *MECP2*, even though they both have same duplication.

## Discussion

In this study, we report two genetically unrelated disorders, 1q21 microdeletion and Xq28 duplication in siblings with intellectual disability. Microarray analysis revealed that the sister, our proband 1, carries 1.24-Mb heterozygous deletion at 1q21.1-1q21.2 region involving 9 genes (Fig. 3a, Table 2) and a 68 Kb deletion within intron 2 of *AUTS2* gene at 7q11.22 (Fig. 2b). 1q21 microdeletion is a contiguous gene deletion syndrome caused by the recurrent distal 1.35-Mb heterozygous deletion in the 1q21 region and characterized by dysmorphic facial features, mild-moderate developmental delay, intellectual disability, microcephaly and short stature [1, 2, 4]. In rare cases, individuals with the deletion may have autism [22], schizophrenia [23], cataracts [2, 5] and isolated heart defects [6]. It is also possible that apparently unaffected patients with 1q21 deletion could have subtle phenotypic features that are highly variable and therefore not easily recognizable. A previous study stated that subtle cataracts and patent ductus arteriosus were detected from one of their patients after directive studies were performed upon discovery of the 1q21 deletion [2]. Although our proband 1 did not have cardiac anomalies and eye problems, a lot of phenotypes detected in proband 1 overlap the common characteristic features of 1q21 deletion syndrome such as microcephaly, developmental delay, mild facial dysmorphism and short stature, suggesting that it is likely that some of the missing genes in chromosome 1 would be the causative genes for her phenotypes, because the deletion in the intron 2 of *AUTS2* gene at 7q11.22 is found in her healthy father as well as the healthy population.

Haploinsufficiency or pathogenic variants of some genes of interest such as *PRKAB2*, *CDH1L*, *BCL9*, *GJA5* and *GJA8* within the region of 1q21 microdeletion likely contributes to the recognizable phenotpyes associated with 1q21 microdeletion. *PRKAB2* is a member of the AMP kinase complex, which maintains systemic and cellular energy homeostasis [24], while *CHD1L* is an enzyme with a role in chromatin modification and DNA damage response [25]. The protein levels of both PRKAB2 and CHD1L were decreased in lymphoblastoid cell lines from persons with 1q21 deletion and AMP kinase function in those LCLs was highly attenuated [26]. In addition, LCLs showed significant reduction in DNA damage repair following treatment with a topoisomerase II inhibitor [26]. These results would support the pathological roles for *PRKAB2* and *CDH1L* in 1q21 microdeletion. Furthermore, the fact that *PRKAB2* is highly expressed in skeletal muscle [27] may explain the short stature and skeletal malformations in patients with 1q21 microdeletion as well as our proband 1. *BCL9* is required for efficient beta-catenin-mediated transcription in Wnt signaling pathway [28], influencing neuroplasticity, adult neurogenesis and mental disorders [29–31]. Therefore, *BCL9* may have contributed to phenotypes including neurological defects and mild intellectual disability in 1q21 microdeletion. Two different gap junction proteins, GJA5 and GJA8 are also associated with phenotypic features in patients with 1q21 microdeletion. GJA5 and GJA8 encode for the cardiac gap junction protein connexin 40 [32] and a transmembrane connexin protein that is necessary for lens growth and maturation of lens fiber cells [33], respectively. Although our proband 1 has neither cardiac abnormalities nor cataract, in many different cases, the phenotypes related with mutations in these two genes have already been reported. GJA5 is thought to be a susceptibility gene for cardiac abnormalities, because one heterozygous nonsense mutation and three missense mutations segregating with this phenotype were found in one and three unrelated families, respectively [34, 35]. Mutations in the GJA8 gene have been found to cause several types of autosomal dominant cataract [36], which have been described as congenital nuclear [37], nuclear pulverulent [38], zonular pulverulent [39], stellate nuclear [39] and posterior subcapsular [40]. Therefore, the most common clinical features of 1q21 microdeletion would have been associated with haploinsufficiency of multiple genes located in the deleted region.

We also describe another individual in the same family, who carries duplication in Xq28 region involving 15 genes and including *MECP2* gene that has been associated with Rett syndrome (Fig. 3c, Table 2). Unlike *MECP2* duplication syndrome, Rett syndrome is caused by the pathogenic *MECP2* variants such as missense mutations and intragenic rearrangements of *MECP2* gene. The pathogenic variants of *MECP2* gene were detected in approximately 80 % of individuals with classic Rett syndrome [41–43] and 40 % of individuals with atypical Rett syndrome [41, 42, 44]. Our proband 2 does not meet clinical criteria for Rett syndrome, but does reflect the clinical features described previously in males with duplication of *MECP2* [45–47]. Among the criteria for the diagnosis of Rett syndrome [48], he only meets evidence of an acquired deceleration in head growth and absent gait. He has lost no skills but is delayed in communication and fine motor skills as well. Among the supportive criteria [48], he has had some falloff in linear growth over the last several months, but otherwise fulfills none of these.

Autism like phenotypes are reported in some cases with Xq28 duplication syndrome [49, 50], and it is very hard to distinguish between Xq28 duplication syndrome and autism, especially children in very young age. Our proband 2, an 8-year old boy, also has a social communication delay and reciprocal behaviors including lack of response to contextual cues, lack of showing and sharing interest, lack of communicative vocalizations with consonant, and repetitive movements with object and body associated with the criteria for autism spectrum disorder [49]. However, the pattern of delays is inconsistent with an autistic spectrum disorder, and his social skills are commensurate with his language and cognitive development whereas autism typically results in more severely delayed social skills versus other areas of development. Although his pattern of behaviors may be characteristic of autism spectrum disorder, it is felt that these behaviors reflect his significant developmental delay and that these characteristics are better accounted for by his developmental delays associated with *MECP2* duplication syndrome.

The *MECP2* gene encodes for MECP2, a chromatin-associated protein that regulates transcription of genes [13, 14]. It is required for maturation of neurons [51] and critical for normal brain function [18]. The presence of an extra copy of the *MECP2* gene is believed to improperly regulate the expression of other genes and this misregulation of gene expression in the brain results in abnormal neuronal function [17, 18], leading to the signs and symptoms of *MECP2* duplication syndrome. Indeed, mild overexpression of Mecp2 in mice causes a progressive neurological disorder including seizures, spasticity and akinesis [17, 52], that are frequently observed in individuals with Xq28 duplication. In addition to *MECP2*, this duplication encompassed several other genes of interest including *L1CAM, FLNA* and *HCFC1* that previously reported in other patients with a Xq28 duplication. L1CAM, a cell-to-cell adhesion molecule, is primarily found in the nervous system and functions in neuronal cell migration and survival [53]. Mutations in *L1CAM* gene are responsible for an X-linked recessive neurological disorder that has been described as X-linked hydrocephalus [54], MASA syndrome [55] or spastic paraplegia type I (SPG1) [56]. FLNA encodes filamin A, an actin-binding protein involved in the reorganization of cytoskeletion to effect in cell migration [57]. Mutations in the X-linked FLNA gene can cause the multiple malformation [58] and neurologic disorder periventricular heterotopia mediated by a failure in neuronal migration into the cerebral cortex [57]. Mutations in the *HCFC1* gene have been found in males with X-linked cobalamin disorder characterized by severe neurological symptoms including intractable epilepsy, seizures and profound development delay, along with variable biochemical manifestations [59]. Another mutation in the 5’ UTR region of the *HCFC1* gene that leads to overexpression of HCFC1 was identified in affected members of a family with X-linked intellectual disability (MRX3, MIM 309541) [60], suggesting that HCFC1 gene may be responsible for some phenotypes, especially intellectual disability, in Xq28 duplication.

**Fig. 5:**
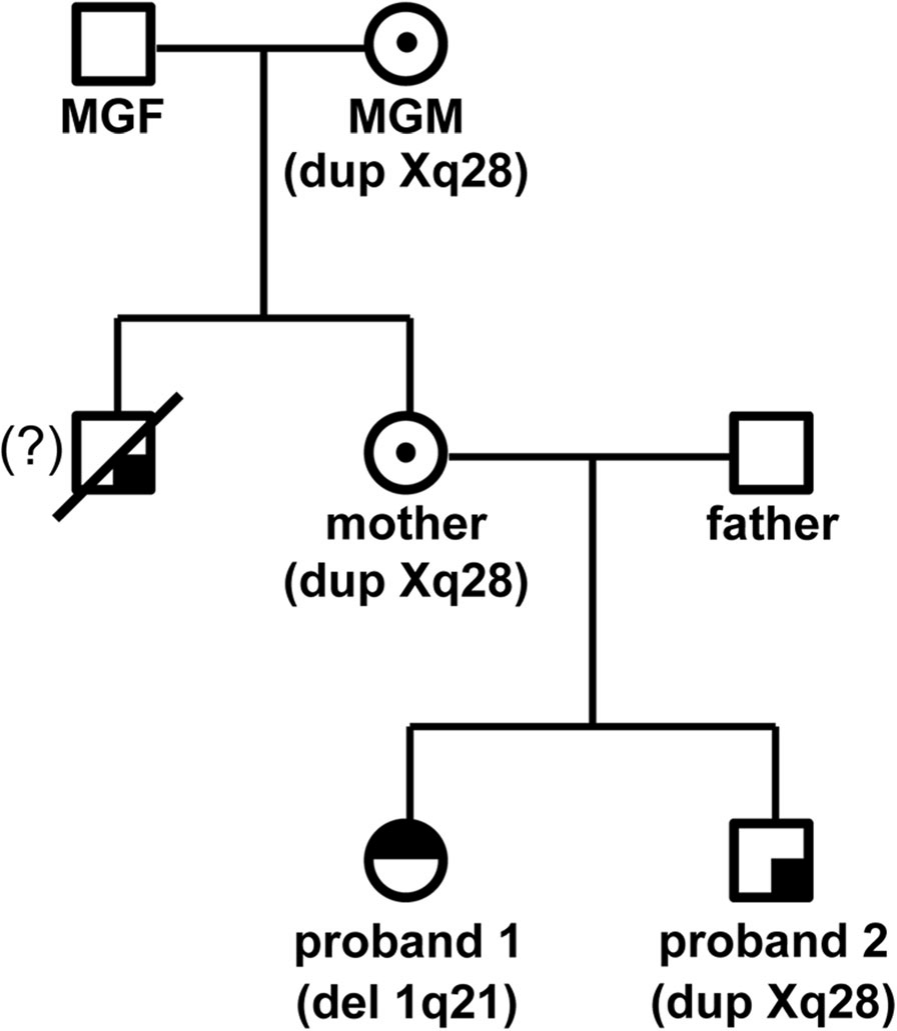
Pedigree of three generations showing the inheritance of 1q21 deletion and Xq28 duplication in the family. 1q21 deletion in proband 1 occurred *de novo* and Xq28 duplication in proband 2 was originally inherited from the maternal grandmother, a carrier who inherited it to his mother, a carrier. The maternal uncle who died at 11 years old from an accident also suffered from a similar phenotype as his nephew. This indicates that the uncle and the nephew might have had the same Xq28 duplication

Xq28 duplication syndrome predominantly affects males and in most cases, it is inherited from a carrier mother, who has favorable skewing of X chromosome inactivation and no symptoms [8]. Using qPCR analysis, the screening of familial inheritance in our study demonstrated that Xq28 duplication in proband 2 was passed down through his mother and maternal grandmother, a carrier with a normal phenotype (Fig. 4c). The RT-qPCR analysis of *MECP2* expression levels revealed that proband 2 has almost double amount of *MECP2* mRNA expression compared to other family members including maternal grandmother and mother who have the same *MECP2* duplication, further suggesting that the skewed inactivation of X chromosome with *MECP2* duplication in two female carriers. This suggests that *MECP2* duplicated on the inactive X chromosome in the asymptomatic carrier females is completely silenced without escaping inactivation. Given that both microdeletion and microduplication of Xq28 encompassing *MECP2* cause common phenotypic features such as intellectual disability, infantile hypotonia, absent speech and developmental regression [18, 45, 47, 61], the dosage of MECP2 is likely stringently regulated, because both decreased and increased amount of MECP2 cause distinct clinical phenotypes. Importantly, proband 2’s maternal uncle who died at 11 years old in a drowning accident had significant intellectual disability and similar phenotypes with proband 2, indicating that maternal uncle was likely affected with the same Xq28 duplication detected in his mother and sister as well as proband 2. Pedigree analysis indicates X-linked recessive inheritance of Xq28 duplication (Fig. 5).

Although patients with 1q21 deletion and *MECP2* duplication syndromes share some phenotypes such as mild dysmorphic features, cardiac abnormalities and mental retardation, it has not been reported for these two genetically unrelated syndromes to occur in the same family, especially in siblings like our case. Therefore, the presence of two seemingly unrelated and rare chromosome abnormalities in siblings is provocative, unusual and calls for further investigations.

## Conclusions

In conclusion, we report on two siblings with genetically unrelated rare syndromes, 1q21 microdeletion or Xq28 duplication. The results of parental CGH and qPCR analyses reveal that the 1q21 microdeletion in the elder sister is *de novo*, and the Xq28 duplication in the younger brother is inherited from the maternal grandmother through the mother. RT-qPCR results revealed that the affected brother has almost double the amount of *MECP2* mRNA expression compared to other family members, including the maternal grandmother and mother with no phenotypes. This indicates the favorable skewing of X-chromosome inactivation in female carriers. Given that the segregation of two rare genetic syndromes in siblings is very unusual, our report draws an attention to the presence of two genetically unrelated syndromes in a same family and further investigations in similar cases would be highly informative for future studies.

## Abbreviations

*ACP6*: Acid phosphatase 6
*AUTS2*: Autism susceptibility candidate 2
*BCL9*: B-cell CLL/lymphoma 9
*CDH1L*: Chromodomain helicase DNA binding protein 1-like
CGH array: Comparative genomic hybridization (CGH) array
CNV: Copy number variation
*FLNA*: Filamin A, alpha
*FMO5*: Flavin Containing Monooxygenase 5
*GJA5*: Gap junction protein, alpha 5
*GJA8*: Gap junction protein, alpha 8
*GPR89B*: G protein-coupled receptor 89B
*HCFC1*: Host cell factor C1
*L1CAM*: L1 cell adhesion molecule
MASA syndrome: Mental retardation, aphasia, spastic paraplegia adducted thumbs syndrome
*MECP2*: Methyl CpG-binding protein 2
*NBPF11*: Neuroblastoma breakpoint family, member 11
*PRKAB2*: Protein Kinase, AMP-Activated, Beta 2 Non-Catalytic Subunit
RT-qPCR: Reverse transcription-quantitative polymerase chain reaction
SPG1: Spastic paraplegia type I
VSD: Ventricular septal defect

## References

[1] Brunetti-Pierri N, Berg JS, Scaglia F, Belmont J, Bacino CA, Sahoo T (2008). Recurrent reciprocal 1q21.1 deletions and duplications associated with microcephaly or macrocephaly and developmental and behavioral abnormalities. Nat Genet.

[2] Mefford HC, Sharp AJ, Baker C, Itsara A, Jiang Z, Buysse K (2008). Recurrent rearrangements of chromosome 1q21.1 and variable pediatric phenotypes. N Engl J Med.

[3] Mefford HC, Eichler EE (2009). Duplication hotspots, rare genomic disorders, and common disease. Curr Opin Genet Dev.

[4] Bernier R, Steinman KJ, Reilly B, Wallace AS, Sherr EH, Pojman N (2015). Clinical phenotype of the recurrent 1q21.1 copy-number variant. Genet Med.

[5] Redon R, Ishikawa S, Fitch KR, Feuk L, Perry GH, Andrews TD (2006). Global variation in copy number in the human genome. Nature.

[6] Christiansen J, Dyck JD, Elyas BG, Lilley M, Bamforth JS, Hicks M (2004). Chromosome 1q21.1 contiguous gene deletion is associated with congenital heart disease. Circ Res.

[7] Sanlaville D, Schluth-Bolard C, Turleau C (2009). Distal Xq duplication and functional Xq disomy. Orphanet J Rare Dis..

[8] Ramocki MB, Tavyev YJ, Peters SU (2010). The MECP2 duplication syndrome. Am J Med Genet A.

[9] El-Hattab AW, Fang P, Jin W, Hughes JR, Gibson JB, Patel GS (2011). Int22h-1/int22h-2-mediated Xq28 rearrangements: intellectual disability associated with duplications and in utero male lethality with deletions. J Med Genet.

[10] Vandewalle J, Van Esch H, Govaerts K, Verbeeck J, Zweier C, Madrigal I (2009). Dosage-dependent severity of the phenotype in patients with mental retardation due to a recurrent copy-number gain at Xq28 mediated by an unusual recombination. Am J Hum Genet.

[11] Vanmarsenille L, Giannandrea M, Fieremans N, Verbeeck J, Belet S, Raynaud M (2014). Increased dosage of RAB39B affects neuronal development and could explain the cognitive impairment in male patients with distal Xq28 copy number gains. Hum Mutat.

[12] Bijlsma EK, Collins A, Papa FT, Tejada MI, Wheeler P, Peeters EA (2012). Xq28 duplications including MECP2 in five females: Expanding the phenotype to severe mental retardation. Eur J Med Genet.

[13] Nan X, Meehan RR, Bird A (1993). Dissection of the methyl-CpG binding domain from the chromosomal protein MeCP2. Nucleic Acids Res.

[14] Nan X, Campoy FJ, Bird A (1997). MeCP2 is a transcriptional repressor with abundant binding sites in genomic chromatin. Cell.

[15] Adler DA, Quaderi NA, Brown SD, Chapman VM, Moore J, Tate P (1995). The X-linked methylated DNA binding protein, Mecp2, is subject to X inactivation in the mouse. Mamm Genome.

[16] D'Esposito M, Quaderi NA, Ciccodicola A, Bruni P, Esposito T, D'Urso M (1996). Isolation, physical mapping, and northern analysis of the X-linked human gene encoding methyl CpG-binding protein, MECP2. Mamm Genome.

[17] Collins AL, Levenson JM, Vilaythong AP, Richman R, Armstrong DL, Noebels JL (2004). Mild overexpression of MeCP2 causes a progressive neurological disorder in mice. Hum Mol Genet.

[18] Gonzales ML, LaSalle JM (2010). The role of MeCP2 in brain development and neurodevelopmental disorders. Curr Psychiatry Rep.

[19] Mayo S, Monfort S, Rosello M, Orellana C, Oltra S, Armstrong J (2011). De novo interstitial triplication of MECP2 in a girl with neurodevelopmental disorder and random X chromosome inactivation. Cytogenet Genome Res.

[20] Fieremans N, Bauters M, Belet S, Verbeeck J, Jansen AC, Seneca S (2014). De novo MECP2 duplications in two females with intellectual disability and unfavorable complete skewed X-inactivation. Hum Genet.

[21] Nishimoto HK, Ha K, Jones JR, Dwivedi A, Cho HM, Layman LC (2014). The historical Coffin-Lowry syndrome family revisited: identification of two novel mutations of RPS6KA3 in three male patients. Am J Med Genet A.

[22] Autism Genome Project C, Szatmari P, Paterson AD, Zwaigenbaum L, Roberts W, Brian J (2007). Mapping autism risk loci using genetic linkage and chromosomal rearrangements. Nat Genet.

[23] Stefansson H, Rujescu D, Cichon S, Pietilainen OP, Ingason A, Steinberg S (2008). Large recurrent microdeletions associated with schizophrenia. Nature.

[24] Ronnett GV, Ramamurthy S, Kleman AM, Landree LE, Aja S (2009). AMPK in the brain: its roles in energy balance and neuroprotection. J Neurochem..

[25] Ahel D, Horejsi Z, Wiechens N, Polo SE, Garcia-Wilson E, Ahel I (2009). Poly(ADP-ribose)-dependent regulation of DNA repair by the chromatin remodeling enzyme ALC1. Science.

[26] Harvard C, Strong E, Mercier E, Colnaghi R, Alcantara D, Chow E (2011). Understanding the impact of 1q21.1 copy number variant. Orphanet J Rare Dis..

[27] Thornton C, Snowden MA, Carling D (1998). Identification of a novel AMP-activated protein kinase beta subunit isoform that is highly expressed in skeletal muscle. J Biol Chem.

[28] de la Roche M, Worm J, Bienz M (2008). The function of BCL9 in Wnt/beta-catenin signaling and colorectal cancer cells. BMC Cancer..

[29] Lambert C, Cisternas P, Inestrosa NC (2015). Role of Wnt Signaling in Central Nervous System Injury. Mol Neurobiol.

[30] Inestrosa NC, Varela-Nallar L (2014). Wnt signaling in the nervous system and in Alzheimer’s disease. J Mol Cell Biol.

[31] Okerlund ND, Cheyette BN (2011). Synaptic Wnt signaling-a contributor to major psychiatric disorders?. J Neurodev Disord.

[32] Lin X, Gemel J, Glass A, Zemlin CW, Beyer EC, Veenstra RD (2010). Connexin40 and connexin43 determine gating properties of atrial gap junction channels. J Mol Cell Cardiol.

[33] White TW, Goodenough DA, Paul DL (1998). Targeted ablation of connexin50 in mice results in microphthalmia and zonular pulverulent cataracts. J Cell Biol.

[34] Yang YQ, Zhang XL, Wang XH, Tan HW, Shi HF, Jiang WF (2010). Connexin40 nonsense mutation in familial atrial fibrillation. Int J Mol Med.

[35] Yang YQ, Liu X, Zhang XL, Wang XH, Tan HW, Shi HF (2010). Novel connexin40 missense mutations in patients with familial atrial fibrillation. Europace.

[36] Shiels A, Mackay D, Ionides A, Berry V, Moore A, Bhattacharya S (1998). A missense mutation in the human connexin50 gene (GJA8) underlies autosomal dominant “zonular pulverulent” cataract, on chromosome 1q. Am J Hum Genet.

[37] Willoughby CE, Arab S, Gandhi R, Zeinali S, Arab S, Luk D (2003). A novel GJA8 mutation in an Iranian family with progressive autosomal dominant congenital nuclear cataract. J Med Genet.

[38] Berry V, Mackay D, Khaliq S, Francis PJ, Hameed A, Anwar K (1999). Connexin 50 mutation in a family with congenital “zonular nuclear” pulverulent cataract of Pakistani origin. Hum Genet.

[39] Polyakov AV, Shagina IA, Khlebnikova OV, Evgrafov OV (2001). Mutation in the connexin 50 gene (GJA8) in a Russian family with zonular pulverulent cataract. Clin Genet.

[40] Devi RR, Vijayalakshmi P (2006). Novel mutations in GJA8 associated with autosomal dominant congenital cataract and microcornea. Mol Vis..

[41] Fukuda T, Yamashita Y, Nagamitsu S, Miyamoto K, Jin JJ, Ohmori I (2005). Methyl-CpG binding protein 2 gene (MECP2) variations in Japanese patients with Rett syndrome: pathological mutations and polymorphisms. Brain Dev.

[42] Li MR, Pan H, Bao XH, Zhang YZ, Wu XR (2007). MECP2 and CDKL5 gene mutation analysis in Chinese patients with Rett syndrome. J Hum Genet.

[43] Zahorakova D, Rosipal R, Hadac J, Zumrova A, Bzduch V, Misovicova N (2007). Mutation analysis of the MECP2 gene in patients of Slavic origin with Rett syndrome: novel mutations and polymorphisms. J Hum Genet.

[44] Kammoun F, de Roux N, Boespflug-Tanguy O, Vallee L, Seng R, Tardieu M (2004). Screening of MECP2 coding sequence in patients with phenotypes of decreasing likelihood for Rett syndrome: a cohort of 171 cases. J Med Genet.

[45] Van Esch H, Bauters M, Ignatius J, Jansen M, Raynaud M, Hollanders K (2005). Duplication of the MECP2 region is a frequent cause of severe mental retardation and progressive neurological symptoms in males. Am J Hum Genet.

[46] del Gaudio D, Fang P, Scaglia F, Ward PA, Craigen WJ, Glaze DG (2006). Increased MECP2 gene copy number as the result of genomic duplication in neurodevelopmentally delayed males. Genet Med.

[47] Friez MJ, Jones JR, Clarkson K, Lubs H, Abuelo D, Bier JA (2006). Recurrent infections, hypotonia, and mental retardation caused by duplication of MECP2 and adjacent region in Xq28. Pediatrics.

[48] Neul JL, Kaufmann WE, Glaze DG, Christodoulou J, Clarke AJ, Bahi-Buisson N (2010). Rett syndrome: revised diagnostic criteria and nomenclature. Ann Neurol.

[49] Peters SU, Hundley RJ, Wilson AK, Warren Z, Vehorn A, Carvalho CM (2013). The behavioral phenotype in MECP2 duplication syndrome: a comparison with idiopathic autism. Autism Res.

[50] Ramocki MB, Peters SU, Tavyev YJ, Zhang F, Carvalho CM, Schaaf CP (2009). Autism and other neuropsychiatric symptoms are prevalent in individuals with MeCP2 duplication syndrome. Ann Neurol.

[51] Nguyen MV, Du F, Felice CA, Shan X, Nigam A, Mandel G (2012). MeCP2 is critical for maintaining mature neuronal networks and global brain anatomy during late stages of postnatal brain development and in the mature adult brain. J Neurosci.

[52] Bodda C, Tantra M, Mollajew R, Arunachalam JP, Laccone FA, Can K (2013). Mild overexpression of Mecp2 in mice causes a higher susceptibility toward seizures. Am J Pathol.

[53] Kenwrick S, Watkins A, De Angelis E (2000). Neural cell recognition molecule L1: relating biological complexity to human disease mutations. Hum Mol Genet.

[54] Schrander-Stumpel C, Fryns JP (1998). Congenital hydrocephalus: nosology and guidelines for clinical approach and genetic counselling. Eur J Pediatr.

[55] Kenwrick S, Jouet M, Donnai D (1996). X linked hydrocephalus and MASA syndrome. J Med Genet.

[56] Fransen E, Van Camp G, Vits L, Willems PJ (1997). L1-associated diseases: clinical geneticists divide, molecular geneticists unite. Hum Mol Genet.

[57] Fox JW, Lamperti ED, Eksioglu YZ, Hong SE, Feng Y, Graham DA (1998). Mutations in filamin 1 prevent migration of cerebral cortical neurons in human periventricular heterotopia. Neuron.

[58] Robertson SP, Twigg SR, Sutherland-Smith AJ, Biancalana V, Gorlin RJ, Horn D (2003). Localized mutations in the gene encoding the cytoskeletal protein filamin A cause diverse malformations in humans. Nat Genet.

[59] Yu HC, Sloan JL, Scharer G, Brebner A, Quintana AM, Achilly NP (2013). An X-linked cobalamin disorder caused by mutations in transcriptional coregulator HCFC1. Am J Hum Genet.

[60] Huang L, Jolly LA, Willis-Owen S, Gardner A, Kumar R, Douglas E (2012). A noncoding, regulatory mutation implicates HCFC1 in nonsyndromic intellectual disability. Am J Hum Genet.

[61] Lugtenberg D, Kleefstra T, Oudakker AR, Nillesen WM, Yntema HG, Tzschach A (2009). Structural variation in Xq28: MECP2 duplications in 1 % of patients with unexplained XLMR and in 2 % of male patients with severe encephalopathy. Eur J Hum Genet.

